# The Role of UFMylation in the Development and Progression of Gastric Cancer

**DOI:** 10.32604/or.2025.066402

**Published:** 2025-10-22

**Authors:** Ying Fang, Anqi Wu, Yu-Sheng Cong, Guoqing Li

**Affiliations:** 1 Department of Gastroenterology, The Second Affiliated Hospital, University of South China, Hengyang, 421001, China; 2 Hunan Provincial Key Laboratory of Basic and Clinical Pharmacological Research of Gastrointestinal Cancer, The Second Affiliated Hospital, University of South China, Hengyang, 421001, China; 3 The Second Clinical Medical School, Hengyang Medical College, University of South China, Hengyang, 421001, China; 4 Department of Clinical Research Center, The Second Affiliated Hospital, University of South China, Hengyang, 421001, China; 5 Key Laboratory of Aging and Cancer Biology of Zhenjiang Province, School of Basic Medical Sciences, Hangzhou Normal University, Hangzhou, 311121, China

**Keywords:** Post-translational modifications, UFMylation, gastric cancer

## Abstract

Gastric Cancer (GC) is a highly prevalent and poorly prognostic gastrointestinal malignancy with low overall treatment efficacy worldwide. Early diagnostic markers and potential therapeutic targets for GC treatment are urgently needed. UFMylation, a novel ubiquitin-like modification is indispensable for numerous fundamental cellular processes. Deficiency in this modification is reported to be associated with several human diseases including cancer. Accumulating evidence suggests that the expression of the key UFMylation components is closely associated with GC cell proliferation, invasion, metastasis, and chemotherapy resistance. Recent clinical studies have further highlighted the prognostic value and therapeutic potential of UFMylation in the clinical management of GC. However, the precise molecular mechanisms through which UFMylation contributes to GC remain largely unclear. This review aims to summarize recent findings on the functional roles of UFMylation in diverse cellular processes, such as endoplasmic reticulum (ER) homeostasis, DNA damage response (DDR), protein translation, and quality control pathways, discuss potential underlying mechanisms in GC development and progression, and to explore potential therapeutic implications targeting the UFMylation pathway in GC.

## 1 Introduction

Globally, gastric cancer (GC) ranks fifth as a cause of cancer-related mortality [1]. As one of the most lethal cancers globally, GC accounts for approximately 970,000 new cases and approximately 770,000 deaths annually [1]. The incidence of GC is particularly high in East Asia, with the top 3 rates reported in Japan (31.6 per 100,000), Korea (27.9 per 100,000), and China (20.6 per 100,000) [2]. The primary risk factors for GC include *Helicobacter pylori* infection, poor dietary habits, and hereditary factors, with higher prevalence observed in men, the elderly, and low-income population [2].

Over the past century, cancer treatment has evolved from surgery and radiation to chemotherapy, endocrine therapy, targeted drugs, and immunotherapies, as well as combinations of these [3]. Despite advancements in modern medicine, early diagnosis of GC remains challenging, which results in poor therapeutic outcomes and low five-year survival rates [2]. Given the incomplete understanding of the pathogenic mechanisms, exploring key molecules and signaling pathways involved in GC development is crucial for elucidating its underlying mechanisms, thereby significantly impacting treatment strategies.

UFMylation is a novel ubiquitin-like modification process through a three-step enzymatic cascade catalyzed sequentially by dedicated E1 Ubiquitin-Like Modifier Activating Enzyme 5 (UBA5), E2 Ubiquitin-Fold Modifier Conjugating Enzyme 1 (UFC1), and E3 enzyme complex consisting of UFM1-Specific Ligase 1 (UFL1) and UFM1-Binding and PCI Domain-Containing Protein 1 (UFBP1) and the Cyclin-Dependent Kinase 5 Regulatory Subunit Associated Protein 3 (CDK5RAP3). UFMylation is a reversible process through de-UFMylation by UFM1-Specific Peptidase 1 (UFSP1) and UFM1-Specific Peptidase 2 (UFSP2). The UFMylation modification is involved in number of cellular processes, such as endoplasmic reticulum (ER) homeostasis, protein synthesis, and DNA damage response (DDR) [4]. Studies suggest that the downregulation of key UFMylation proteins, such as Ubiquitin-Fold Modifier 1 (UFM1), UFBP1, and CDK5RAP3, is closely associated with GC cell proliferation, invasion, and chemotherapy resistance, and multiple signaling pathways are involved, including Wnt/β-catenin, and Phosphatidylinositol 3-Kinase/Protein Kinase B (PI3K/AKT) [5,6]. Here, we outline the role of UFMylation in GC to provide new insights into the understanding of GC development and progression and to explore potential therapeutic strategies based on this modification.

## 2 Molecular Classification and Therapeutic Advances in Gastric Cancer

GC is a malignant digestive tract tumor with significant molecular heterogeneity and complex pathological types. Histologically, it is generally divided into three subtypes: intestinal, diffuse, and mixed [7]. In recent years, with deeper insights into the molecular pathology of GC, The Cancer Genome Atlas (TCGA) project has proposed four new molecular subtypes: Epstein–Barr virus-positive (EBV), microsatellite instability (MSI), genomically stable (GS), and chromosomal instability (CIN) [8]. These subtypes exhibit distinct molecular mechanisms, clinical characteristics, and therapeutic strategies, thereby adding complexity to the diagnosis and treatment of GC [9].

Endoscopic assessment and histological biopsy are the golden standard for GC diagnosis [9]. However, early-stage GC often presents with nonspecific or minimal symptoms, and may lack reliable biomarkers, making timely detection difficult. The primary treatment for early-stage GC is surgical resection. Early-stage GC has a five-year surgical success rate of over 90%, which is significantly higher than that of advanced disease [10]. The effectiveness of these operations are limited because early-stage GC lacks obvious symptoms, and most people are diagnosed at an advanced level. In such cases, chemotherapy is the main approach for advanced GC, a combination of fluoropyrimidines and platinum-based agents is used as first-line chemotherapy, while paclitaxel and irinotecan serve as second-line treatment options [11]. Although chemotherapy extends patient survival to some extent, the development of chemoresistance and adverse effects is still a significant challenge. Therefore, recent studies have focused on the molecular mechanisms of chemoresistance and strategies to improve the efficacy of clinical outcome [12,13].

For advanced GC, targeted therapy and immunotherapy are also becoming more important. Trastuzumab is a mono antibody that targets human epidermal growth factor receptor 2 (HER2), and it has been shown to significantly boost overall survival in patients with HER2-positive metastatic GC [14]. Furthermore, programmed cell death protein 1/programmed death-ligand 1 (PD-1/PD-L1) pathway inhibitors, such as pembrolizumab and nivolumab, have demonstrated significant efficacy in patients with EBV+, high microsatellite instability (MSI-H) or a high tumour mutation burden (TMB) [15]. In addition, with the continuous development of medical research, the exploration of novel prognostic biomarkers such as ferroptosis-related long non-coding RNAs and N^6^-methyladenosine modifications has also provided new directions for GC treatment [16,17]. Meanwhile, with the advancement of research, several novel therapeutic strategies for gastric cancer have emerged. For example, triptolide induces reactive oxygen species (ROS) accumulation by directly binding to peroxiredoxin 2 (PRDX2), leading to both apoptosis and cytoprotective autophagy in gastric cancer cells [18]. It has been shown that various key components of UFMylation are correlated with the stage of GC and play a critical role in the progression and chemoresistance of GC, such as UFM1 [5], UFBP1 [19] and CDK5RAP3 [20,21]. More importantly, UFMylation has also been involved in the tumor immune evasion via UFMylation of immune checkpoint proteins PD-1/PD-L1 [22–24], which indicated an potential role of UFMylation in the chemotherapy and immunotherapy of GC, and may be potential novel therapeutic targets of GC.

## 3 The Mechanisms of UFMylation

Protein post-translational modification (PTM) encompasses a series of chemical alterations that occur after protein biosynthesis. PTMs such as ubiquitin (Ub) or ubiquitin-like modifiers (UBLs) (e.g., SUMO, NEDD8, ISG15, URM1, and UFM1) regulate a wide range of essential cellular processes, including transcription, replication, cell cycle control, signal transduction, DDR, and apoptosis [25].

**Figure 1:**
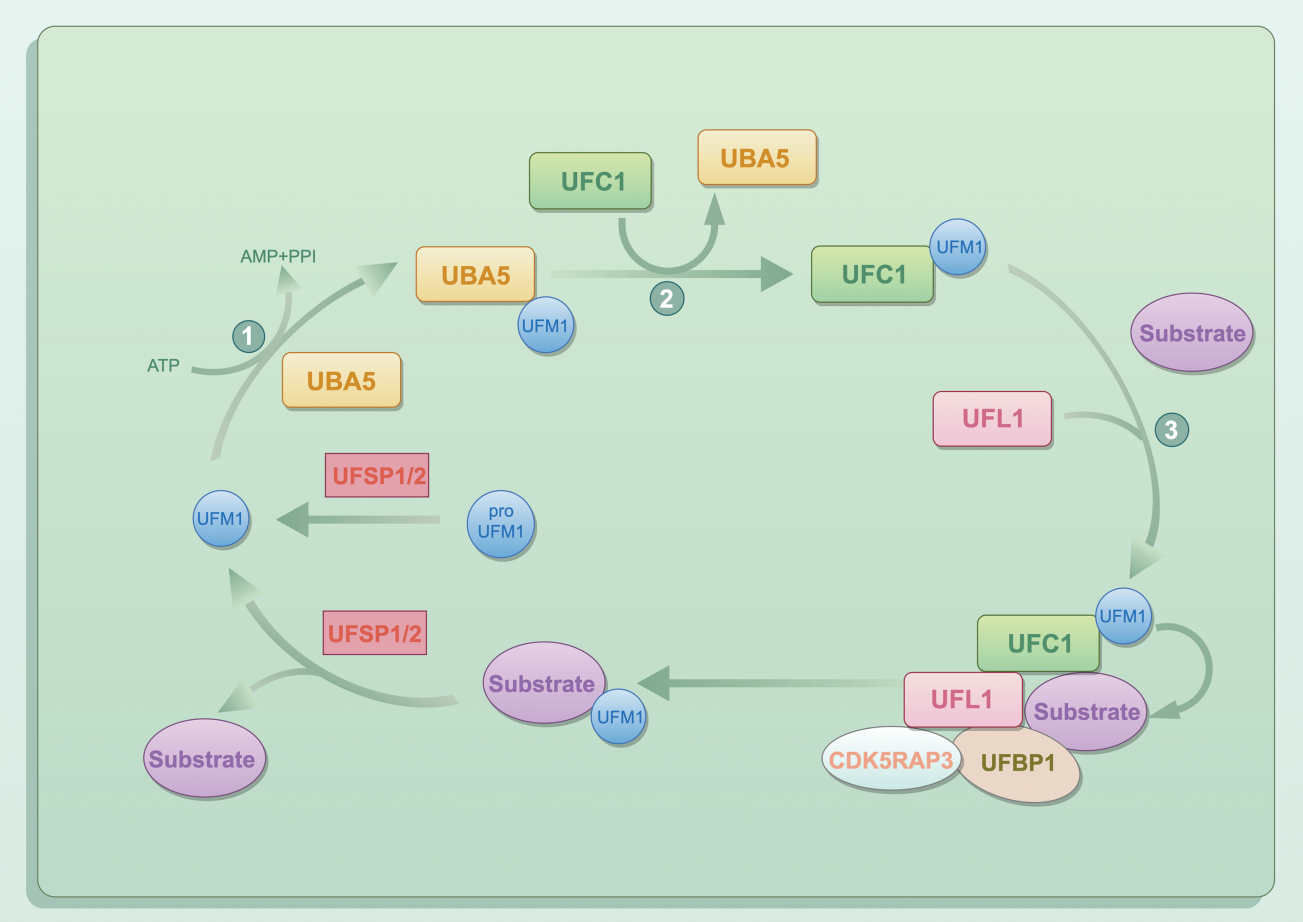
The conjugation pathway of Ubiquitin-Fold Modifier 1 (UFM1). Created using Figdraw V2.0 (https://www.figdraw.com). UFM1, ubiquitin-fold modifier 1; UFSP1/2, UFM1-specific peptidase 1/2; UBA5, ubiquitin-like modifier activating enzyme 5; UFC1, ubiquitin-fold modifier conjugating enzyme 1; UFL1, UFM1-specific ligase 1; UFBP1, UFM1-binding and PCI domain-containing protein 1; CDK5RAP3, cyclin-dependent kinase 5 regulatory subunit-associated protein 3

The majority of UBLs show limited sequence similarity to ubiquitin. Nevertheless, they share comparable tertiary structures and attach covalently to target proteins through E1-E2-E3 enzymatic reactions similar to ubiquitination. UFM1 is one of the most recently identified UBLs [26]. UFMylation is a multistep enzymatic process akin to ubiquitination but possesses unique characteristics. The UFM1 precursor is first cleaved into mature and activated UFM1 by the specific protease UFSP1/2, and then conjugated to the target protein through a three-step enzymatic reaction, as shown in Fig. 1. UFM1 is adenylated by UBA5 through an ATP-dependent reaction, forming a high-energy thioester bond with cysteine residue (Cys250) of UBA5. Subsequently, activated UFM1 is transferred to UFC1, establishing a similar high-energy thioester linkage. Finally, under the catalysis of the E3 ligase UFL1, UFM1 is transferred from UFC1 to specific lysine residues on substrate proteins, completing the UFMylation modification. Concurrently, the specific proteases UFSP1 and UFSP2 can excise UFM1, thereby regulating the dynamic balance of UFMylation [27].

There are some structural differences in the enzymes of UFMylation when compares to other UBLs parallelly. In contrast to other E1 in UBLs, the adenylation activity and catalytic cysteine (Cys250) of UBA5 is located on a single domain rather than two separate domains [28]. Compares to other E2 in UBLs, the UFC1 lacks the conserve catalytic histidine–proline–asparagine (HPN) motif, instead it is replaced by a threonine–alanine–lysine (TAK) motif [29]. And UFC1 does not have strong homology with other known E2s [30]. At last, the UFL1 lacks neither RINGs or HECTs domain like majority E3s in UBL do [31]. Furthermore, UFMylation is primarily localized in the ER, suggesting a unique role in ER-related biological functions, such as ER homeostasis. These specific functions of UFMylation are closely linked to stress responses and may contribute to GC, where ER stress and disrupted protein homeostasis are common [32].

## 4 The Functions of UFMylation

### 4.1 DNA Damage Response

UFMylation is involved in DDR processes to maintain genome stability. Ataxia-Telangiectasia Mutated (ATM) is a serine/threonine protein kinase essential for initiating and regulating the DDR [33]. When DNA double-strand breaks (DSBs) occur, ATM rapidly activates, initiating DNA damage signalling cascades by phosphorylating downstream target proteins such as Nijmegen breakage syndrome protein 1 (NBS1). The MRN complex—composed of meiotic recombination 11 homolog 1 (MRE11), RAD50 double strand break repair protein (RAD50), and NBS1—facilitates the selection and translation of ATMs to damaged sites by detecting DSBs quickly and acting as a DNA damage sensor. In this process, MRE11 undergoes UFMylation at lysine residue 282, which promotes efficient MRN complex assembly and enhances ATM kinase activation [34]. At the DNA damage sites, the UFMylation-specific E3 ligase UFL1 is recruited by the MRN complex, where it promotes histone UFMylation, playing an important regulatory role in the DDR. Particularly, UFL1 facilitates the UFMylation of histones, which subsequently promotes the recruitment of the methyltransferase Suv39h1. Suv39h1 catalyzes the formation of histone H3 lysine 9 trimethylation (H3K9me3), creating a chromatin environment conducive to DNA restoration. The histone acetyltransferase Tip60, which acetylates ATM enzyme at lysine 3016 (K3016), further increase ATM enzyme activity and boost DDR signalling. In addition to, activated ATM phosphorylates UFL1 at serine residue 462, which enhances UFL1’s E3 ligase activity, developing a positive feedback loop that strengthens the DNA damage repair answer [35].

### 4.2 Endoplasmic Reticulum Homeostasis

UFMylation modifies newly synthesized proteins on the ER. This modification assists in ensuring correct protein folding and maintaining cellular protein homeostasis. In response to external stimuli, UFMylation regulates the unfolded protein response (UPR). This modulation helps cells alleviate ER stress and subsequently restore the normal physiological function of the ER. The study by Liu et al. found that depletion of UFBP1 leads to apoptosis and elevated ER stress. UFBP1 interacts with the kinase domain of inositol-requiring enzyme 1 alpha (IRE1α), regulating its protein stability. This regulatory interaction depends on the UFMylation modification at the K267 residue of UFBP1 [36].

### 4.3 Other Cellular Functions

UFMylation is involved in the regulation of cellular homeostasis. For example, the deletion of the UFMylation enzyme genes *UBA5* and *UFL1* and the related articulating protein *UFBP1* in mice reveals the necessity of the UFMylation enzymes for cellular development and differentiation [37,38]. Furthermore, UFMylation is associated with processes such as autophagy, transcriptional regulation, and signal transduction. UFL1 is recruited by UFBP1 to the endoplasmic reticulum, where it associates with Ribophorin (RPN1) and Ribosomal Protein L26 (RPL26) to regulate ER-specific autophagy [39]. The loss of Low-density lipoprotein receptor-related protein 1 (LRP1) has been shown to promote hepatocellular carcinoma progression through UFL1-mediated activation of NF-κB signaling [40]. These functions collectively highlight its essential role in maintaining cellular homeostasis.

## 5 Dysregulated UFMylation in Cancer

Currently, only around a dozen substrates for UFMylation have been identified. Among these substrates, two have been closely related to cancer, Activating Signal Cointegrator 1 (ASC1) and p53. ASC1 is a nuclear receptor transcriptional co-activator containing multiple UFMylation sites, including lysine residues K324, K325, K334, and K367. ASC1 undergoes UFMylation upon estradiol (E2) stimulation [41]. It binds to estrogen receptor α (ERα), which recruits transcriptional co-enhancers such as p300 and SRC1 to the promoter of ERα targets [42]. This connection dramatically enhances transcriptional activation of ERα target genes, including Cyclin D1, c-Myc, and pS2, leading to increased proliferation of breast cancer cells and finally promoting breast cancer progression [41]. p53, a tumor suppressor protein, can be covalently modified by UFM1. Deletion of key UFMylation regulators such as UFL1 or UFBP1 destabilizes p53, ultimately promoting cell growth and tumor formation. Liu et al. demonstrated that UFMylation is an important post-translational modification involved in regulating the stability of the tumor suppressor protein p53 [43]. These two examples indicate UFMylation play different roles in different cancer types when interacting with different substrates.

In addition to the UFMylation substrate research, abnormal of UFMylation components also plays as tumor suppressors in some cancer types but promote tumor progression in others. In GC, both UFM1 and UFBP1 play a tumor suppressing role. Lin et al. found that UFM1 suppresses GC cell invasion and metastasis by decreasing 3-phosphoinositide-dependent protein kinase-1 (PDK1) expression, thereby inhibiting the AKT/glycogen synthase kinase-3 beta (GSK3β) signaling pathway and reducing epithelial-mesenchymal transition (EMT) activity [5]. Another study showed that elevated UFBP1 expression correlates with prolonged progression-free survival in patients with advanced GC receiving platinum-based chemotherapy. Moreover, the study revealed that UFBP1 increased GC cell sensitivity to cisplatin through moinhibitors of UFSP2 may boostdulation of the nuclear factor erythroid 2–related factor 2/aldo-keto reductase family 1 member C (Nrf2/AKR1C) signaling axis [19]. Similar to GC, CDK5RAP3 and UFL1 also play a tumor suppressing role in renal carcinoma and melanoma, respectively. Compared with adjacent non-tumor tissues, CDK5RAP3 expression is significantly downregulated in renal carcinoma. As a potential tumor suppressor, it plays a key role in renal carcinoma by regulating autophagy [44]. In melanoma patients, low levels of UFL1 expression in tumor tissues are significantly associated with poor responses to anti-PD-1 immunotherapy [24]. Analysis of the TCGA database reveals that approximately 20% of prostate adenocarcinoma cases exhibit copy number loss of key UFMylation genes (UFM1 or UFL1), suggesting potential dysfunction of the UFMylation pathway in prostate cancer [27].

In contrast, in some cancers, high expression of certain UFMylation-associated proteins promotes tumor growth and invasion. For example, UBA5 is highly expressed in lung adenocarcinomas and acts as a proto-oncogene. It enhances cell growth, increases cisplatin resistance, and promotes immune escape, thereby facilitating tumor progression and chemotherapy resistance [45]. Higher expression of UFM1 was found in oral squamous cell carcinoma (OSCC), and its overexpression was associated with shorter overall survival, suggesting that UFM1 may be a poor prognostic factor for OSCC [46]. CDK5RAP3 is highly expressed in clinical specimens of neuroblastoma (NB) and correlates with poor prognosis in NB patients. It was also found that CDK5RAP3 promoted NB cell growth and development both *in vivo* and *in vitro* [47]. In addition, it was found that in response to 17β-estradiol (E2) stimulation, ERα binds to the nuclear receptor coactivator ASC1, leading to the UFMylation of ASC1, which promotes the development of breast cancer [41]. UFSP2 exhibits frequently copy-number alterations in colon cancer, and its low expression leads to elevated levels of UFMylation, promoting tumor cell proliferation [48]. UFMylation of ribosomal protein L10 (RPL10)in pancreatic cancer tissues enhances cell proliferation and promotes cancer cell stemness by upregulating Krüppel-like factor 4 (KLF4) expression [49]. Moreover, studies have shown that inhibiting UBA5 activity can effectively reduce the tumorigenic potential of pancreatic cancer cells. Thus, UBA5 may serve as a novel therapeutic target for pancreatic cancer [50]. In glioblastoma stem cells, knockout of all core components of the UFMylation pathway (such as UBA5, UFC1, UFL1, and UFSP2) significantly reduces cell viability. Similarly, treatment with a UBA5 inhibitor markedly suppresses the proliferation of glioma stem cells [51].

These observations suggest a complex role of UFMylation in cancer, which may act as either an oncogene or a tumor suppressor, depending largely on the specific cancer type and cellular environment. It has been shown that UFMylation components suppress tumor progression in GC, renal carcinoma and melanoma, but promote tumor progression in lung adenocarcinomas, neuroblastoma, breast cancer, pancreatic cancer and glioblastoma. This dual role suggests the need to further study the tumor-specific regulation of UFMylation, especially in GC where its tumor-suppressive effect is more evident. A better understanding of these differences may support the development of targeted therapies and help avoid cross-reactivity in treatments of multiple cancer types.

## 6 UFMylation Components and Gastric Cancer

In recent years, UFMylation-related proteins have been shown to play a key role in the development and progression of GC. Analysis of TCGA and Gene Expression Omnibus (GEO) databases revealed that CDK5RAP3 expression is generally low in GC tissues and that its reduced expression is closely associated with malignant features—including larger tumor size, deeper infiltration and advanced Tumor–Node–Metastasis (TNM) staging—as well as significantly lower patient survival rates [21]. Immunohistochemical analysis further confirmed that CDK5RAP3 expression is significantly lower in GC tissues compared to normal tissues and is negatively correlated with the cancer stem cell (CSC) marker CD44. Patients exhibiting high CDK5RAP3 expression have a better prognosis, whereas those with low CDK5RAP3 and high CD44 expression experience the poorest survival rates, suggesting that CDK5RAP3 may modulate the characteristics of CSCs [52]. Further studies revealed that CDK5RAP3 may inhibit GC cell proliferation by regulating the nuclear localization of the DNA replication-associated protein minichromosome maintenance complex component 6 (MCM6). When CDK5RAP3 expression is low, MCM6 accumulates abnormally in the nucleus and promotes cell proliferation, suggesting that CDK5RAP3 functions as a tumor suppressor by regulating cell cycle progression [53].

Similarly, UFBP1 expression is significantly reduced in GC tissues compared with adjacent non-tumor tissues. This low expression is closely associated with poor tumor differentiation and advanced TNM staging, indicating that UFBP1 might play a key role in GC progression [20]. Moreover, elevated UFBP1 expression in GC patients is associated with improved progression-free survival (PFS) after platinum-based chemotherapy [19]. Similarly, UFM1 expression is also low in GC tissues. its reduced expression is closely linked to advanced TNM stage and poor survival. Patients exhibiting high UFM1 expression have significantly higher five-year survival rates compared to those with low expression levels [5].

**Figure 2:**
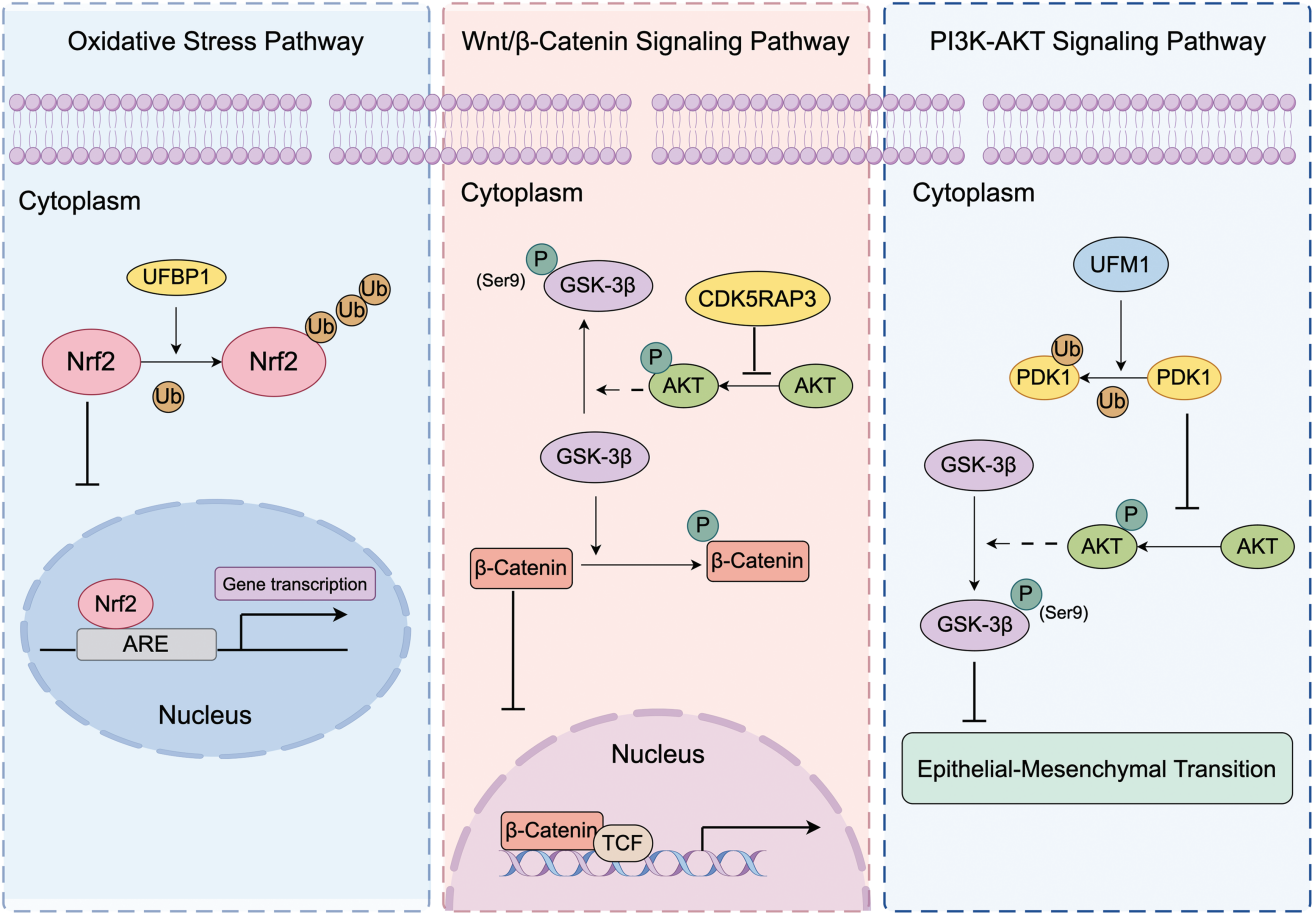
The indirect regulation of gastric cancer development by UFMylation. Created using Figdraw V2.0 (https://www.figdraw.com). Nrf2, nuclear factor erythroid 2–related factor 2; Ub, ubiquitin; ARE, antioxidant response element; UFBP1, UFM1-binding and PCI domain-containing protein 1; GSK-3β, glycogen synthase kinase-3 beta; CDK5RAP3, cyclin-dependent kinase 5 regulatory subunit-associated protein 3; AKT, protein kinase B; β-catenin, beta-catenin; TCF, T-cell factor; UFM1, ubiquitin-fold modifier 1; PDK1, 3-phosphoinositide-dependent protein kinase-1

Although the specific molecular mechanisms remain incompletely understood, evidence suggests that UFMylation components may be directly involved in gastric progression through the regulation of multiple signaling pathways, as shown in Fig. 2.

### 6.1 Oxidative Stress Pathway (Nrf2-Related Signaling)

UFBP1 is a key component of the UFMylation system. Binding directly to and co-localizing with Nrf2 in the cell, UFBP1 reduces Nrf2 protein amounts by supporting K48-linked ubiquitination of Nrf2, thereby enhancing its degradation [19,54]. This regulation suppresses the expression of downstream antioxidant genes, such as members of the AKR1C family, by significantly altering Nrf2-mediated regulatory action of antioxidant response elements (AREs) [55]. In response, oxidative stress and chemotherapeutic agents are more sensitive to cell due to this restriction [56]. UFBP1 regulates Nrf2 at the post-transcriptional level through the ubiquitin-proteasome system (UPS), without affecting its mRNA expression. When cells are treated with proteasome inhibitors such as MG132 or Btz, the degradative effect of UFBP1 on Nrf2 is significantly suppressed [19]. The study by Hu et al. underscored the potential of UFBP1 as a prognostic marker for GC and offered a novel therapeutic method for overcoming chemotherapy resistance [19].

### 6.2 Wnt/***β***-Catenin Signaling Pathway

In the development of GC, the Wnt/β-Catenin signaling pathway is crucial in regulating cell proliferation and migration. In particular, GSK-3β, a key negative regulator of this pathway, promote phosphorylation and proteasomal degradation of β-catenin which leads to a decreased transcriptional activation of downstream T-cell factor (TCF)/ Lymphoid enhancer-binding factor 1 (LEF1) [57]. Activated AKT phosphorylates results in inhibition of GSK-3β and activation of the Wnt signaling pathway. Overexpression of CDK5RAP3 in GC significantly reduces AKT phosphorylation, which in turn decreases GSK-3β phosphorylation at Ser9 and thereby restores GSK-3β activity [6]. This effect markedly diminishes both the level and activity of nuclear β-catenin. Conversely, knockdown of CDK5RAP3 significantly increases AKT phosphorylation, leading to elevated p-GSK-3β levels. Moreover, using siRNA to reduce AKT expression reverses the increased levels of p-GSK-3β and β-catenin that are triggered by reduced CDK5RAP3 expression. This indicates that in GC, CDK5RAP3’s regulation of the Wnt/β-catenin pathway is dependent on AKT [6,58].

### 6.3 PI3K-AKT Signaling Pathway

EMT is a critical biological process that facilitates cancer cell invasion and metastasis [59]. Lin et al. demonstrated that overexpression of UFM1 effectively prevents EMT by increasing E-cadherin expression and reducing the levels of N-cadherin and vimentin. Further studies revealed that UFM1 knockdown in GC cells results in a significant elevation of phosphorylated AKT (p-AKT) levels [5]. Moreover, UFM1 inhibits GC metastasis in a 3-phosphoinositide-dependent protein kinase-1 (PDK1)-dependent manner. PDK1 is a key upstream kinase of the PI3K/AKT signaling pathway that activates AKT and inhibits GSK3β [60]. The activity of the PDK1 is directly linked to EMT. Under UFM1 knockdown conditions, the invasion and migration of GC cells are significantly enhanced. Moreover, following siRNA-mediated interference with PDK1 expression, the increased invasion and migration induced by UFM1 depletion were reversed. Mechanistically, UFM1 binds to PDK1 and promotes its ubiquitination, which leads to reduced PDK1 expression. This decrease in PDK1 levels subsequently inhibits the phosphorylation of AKT and GSK-3β [5,61]. This process effectively inhibits EMT in GC cells and limits their migration and invasion, thereby exerting an indirect anticancer effect.

Taken together, UFM1, CDK5RAP3, and UFBP1 are directly involved in gastric carcinogenesis and progression through the regulation of multiple signaling pathways. Clinical data further support these findings, as GC patient samples generally exhibit downregulation of UFM1, UFBP1, and CDK5RAP3 expression, with low expression levels correlating with poorer prognosis and higher metastasis rates [20,52,58]. These results suggest that UFMylation components play a critical role in the development of GC.

## 7 UFMylation and Tumor Immune Evasion: Implications for Gastric Cancer

Immune evasion is one of the key mechanisms by which tumor cells promote their growth and metastasis by avoiding immune system attacks. In this process, the aberrant activation of the PD-1/PD-L1 pathway plays a major role in tumor immune evasion. Recent studies indicate that UFMylation contribute significantly to immune evasion mechanisms. It regulates the stability of PD-L1, directly affecting the immune evasion mechanisms of tumor cells. This process reduces PD-L1 accumulation on the tumor cell surface and weakens its suppression of effector T cells [24]. Additionally, UFMylation maintains PD-1 stability and increases anti-tumor immune function in T cells [22]. This bidirectional regulation gives UFMylation the potential to enhance anti-tumor immune responses.

### 7.1 UFMylation in Tumor Cell Immune Evasion

According to the TCGA database, UFL1 gene expression is typically downregulated across different cancer types. For instance, a melanoma patient with low UFL1 expression is closely associated with high PD-L1 expression [24]. This is linked to an immunosuppressive tumor microenvironment, and a poor response to anti-PD-1 therapy. This is consistent with the fact that PD-L1 is a substrate of UFMylation. UFL1 mediates UFMylation to specific lysine residues of PD-L1 synergistically promotes PD-L1 ubiquitination and directs its degradation through the proteasome [24]. Since PD-L1 is a crucial immune checkpoint molecule, its high expression on tumor cells interacts with PD-1 on T cells, preventing T cell anti-tumor functions and supporting tumor immune evasion [62]. In triple-negative breast cancer, placenta-specific 8 (PLAC8) stabilizes PD-L1 through UFMylation, thereby suppressing immune cell activity [23]. This process enhances PD-L1–mediated immune evasion and promotes tumor proliferation. Increasing PD-L1 UFMylation considerably reduces its steady-state levels, thereby attenuating its immune suppressive effects. Studies show that inhibitors of UFSP2 may boost PD-L1 UFMylation levels and promote its degradation. This process demonstrates significant anti-tumour immune effects in mouse tumour models [24].

### 7.2 Role of UFMylation in T Cell Immunity

UFMylation plays a vital role in T cells by regulating PD-1 stability, thereby influencing anti-tumor immune responses. UFL1 catalyzes the UFMylation of PD-1 in T cells, which prevents its ubiquitination. As a result, PD-1 is protected from proteasomal degradation, and its stability is maintained [22,43]. The absence of UFL1 results in reduced PD-1 UFMylation in T cells, contributing to the PD-1 degradation, which enhances T cell anti-tumor function. Similar to PD-1 blockade, mice with UFL1 deficient T cells exhibit anti-tumour effects, including significantly reduced PD-1 levels and enhanced T-cell immune responses [22]. Additionally, UFL1 deficiency significantly increases the effectiveness of anti–cytotoxic T-lymphocyte–associated protein 4 (anti-CTLA-4) treatment, suggesting that combining the UFMylation pathway with conventional immune checkpoint therapies may have a synergistic result [63]. Therefore, UFL1 is a potential immune-enhancing target that warrants further exploration in tumor immunotherapy.

### 7.3 Potential Role of UFMylation in Immune Evasion in Gastric Cancer

Recently, immune checkpoint inhibitors like anti-PD-1 and anti-PD-L1 antibodies have become effective therapeutic strategies for various cancers [64,65]. In GC, PD-1/PD-L1 inhibitors such as nivolumab and pembrolizumab have shown promising clinical efficacy in some patients [63,66]. Although direct evidence is currently limited, UFMylation may play a role in immune evasion in GC. This hypothesis is based on observed similarities in the immune microenvironment and checkpoint architecture between GC and other malignancies. UFMylation may affect the stability of PD-1/PD-L1 or modulate T cell function, thereby influencing the tumor immune landscape. However, these assumptions require further experimental validation to clarify the specific role of UFMylation in immune regulation. Elucidating this mechanism could enhance our understanding of tumor immune escape and support the development of new immunotherapeutic approaches.

## 8 Pharmacological Intervention Targeting UFMylation

In recent years, some small molecules that modulate the UFMylation pathway have been identified in preclinical experiments, creating new opportunities for targeted cancer therapy. Among these, UBA5, the E1 enzyme in the UFMylation cascade, has consequently emerged as a promising drug target. In early studies, da Silva et al. developed a noncompetitive UBA5 inhibitor compound 8.5 that was based on an adenosine–zinc(II) cyclen scaffold [67]. This inhibitor specifically interacts with the UBA5 ATP pocket, particularly targeting residues Glu209 and Asp183. Compound 8.5 exhibits an IC_50_ value of 4.0 μM against UBA5 *in vitro*, with more than 20-fold selectivity over other E1 enzymes. It also selectively inhibits the proliferation of Sk-Luci6 tumor cells, which exhibit high UBA5 expression. While another independent study showed that the covalent ligand DKM 2-93 can specifically binds the catalytic cysteine residue (Cys250) of UBA5, thereby inhibiting UFMylation and significantly reducing pancreatic cancer cell viability *in vitro* and *in vivo* [50]. Furthermore, the natural product Usenamine A from the lichen *Usnea longissimi* has also been reported to exhibit potential inhibitory activity against UBA5 [68]. In breast cancer cells, Usenamine A reduces UBA5 expression, thereby inducing ER stress and autophagy. It also markedly inhibits cell proliferation and invasion. Molecular docking analyses further suggest that Usenamine A likely exerts its inhibitory effect by binding to interface between UFM1 and UBA5 rather than to the ATP pocket. In addition, other study have reported identification of five novel selective UBA5 inhibtors by a high-throughput screening (HTS) systems. These inhibitors effectively block UFMylation both *in vitro* and in cells, providing promising candidates for UBA5-targeted therapy [69].

Additionally, UFSP2 has also been recognized as a potential therapeutic target. Through virtual screening, researchers identified a covalent small-molecule inhibitor called compound-8. This molecule inhibits the protease activity of UFSP2, thereby enhancing the UFMylation of PD-L1 and promoting its degradation. It also significantly improves the efficacy of anti-PD-1 therapy in mouse tumor models [24].

Moreover, recent studies indicate that AMP-activated protein kinase (AMPK)-mediated phosphorylation of UFL1 at Thr536 suppresses its interaction with PD-1, thereby reducing the UFMylation of PD-1 and promoting its degradation. In mouse models, administration of AMPK activators such as metformin or A-769662 disrupts the UFL1–PD-1 interaction and enhances T-cell antitumor function [22]. These findings suggest that indirect regulation of UFL1 activity through AMPK activators may represent an adjuvant therapeutic strategy with immunostimulatory effects.

Although these pharmacological strategies have not yet been investigated in gastric cancer models, the role of UFMylation in regulating PD-L1 stability and immune evasion has been demonstrated in other tumor types. This evidence provides a strong theoretical foundation for their potential application in gastric cancer. Pharmacological targeting of the UFMylation has shown promise in both tumor regulation and immune modulation, demonstrating its therapeutic potential for dual intervention.

## 9 Perspectives

GC is among the most prevalent malignancies and ranks fifth as a cause of cancer-related mortality [1]. Early-stage GC lacks obvious symptoms, so most of cases are diagnosed at an advanced level [70]. Currently, the main treatments for advanced GC include chemotherapy, targeted therapy and immunotherapy [71]. However, these treatments have problems such as chemotherapy resistance and lacks of effective targets, resulting in limited therapeutic efficacy [72]. Therefore, the study of key molecules and signalling pathways involved in GC progression is crucial for the diagnosis and therapeutic strategies of GC.

The current study suggests that the essential components of UFMylation (such as UFM1, UFBP1, and CDK5RAP3) are underexpressed in GC tissues, and this downregulation is closely linked with increased cell proliferation, invasion, drug resistance, and poor patient prognosis [20,52,58]. Through the regulation of various signaling pathways, it has been suggested that UFMylation may be critically involved in GC [5,6,19]. However, the specific molecular mechanisms remain unclear, especially the regualtory mechanisms of key enzymes (E1, E2, and E3) and their substrate modifications, which need further investigation.

Moreover, UFMylation may contribute to immune evasion in GC through its regulation of the PD-1/PD-L1 immune checkpoint pathway. Clarifying the role of UFMylation in GC immunity, particularly in combination with immune checkpoint inhibitors, could provide valuable insights for enhancing immunotherapeutic strategies. Recently, the identification of small molecules such as compound 8.5 [67], DKM 2–93 [50], Usenamine A [68], compound-8 [24], and AMPK activators [22] indicate that research targeting the UFMylation pathway is moving from basic research toward translational applications. However, further studies are needed to comprehensively evaluate the potential toxicity, off-target effects on UBL effects, and pharmacokinetic profiles of these inhibitors before clinical application.

Currently, most research is limited to *in vitro* cellular models, lacking direct validation of the UFMylation pathway in clinically relevant models, such as immunohistochemical (IHC) scoring of UFMylation pathway components in large GC patient cohorts. Thus, future studies should incorporate patient-derived xenograft (PDX) models, which maintain patient-specific histological and molecular features, offering a more faithful tumor microenvironment than conventional cell-line xenografts.

Additionally, novel approaches such as disrupting tumor–macrophage crosstalk via small extracellular vesicles (EVs) demonstrate therapeutic potential. Especially in cancer immunotherapy, EVs are expected to be developed as drug delivery vehicles or immunomodulatory tools [73]. Future research should expand to include cellular engineering and tumor microenvironment modulation techniques to systematically explore the role of UFMylation in multidimensional immune regulation networks. Furthermore, the combined application of UFMylation modulators with standard chemo or HER2-targeted therapy warrants further investigation, potentially promoting the development of innovative GC treatment strategies.

Open questions remain, including what determines substrate specificity of UFMylation in different tissues, and whether GC contains UFMylation targets beyond the usual suspects. Addressing these gaps will be essential for advancing the field.

## Data Availability

Not applicable.
